# Occupational medical prophylaxis for the musculoskeletal system: A function-oriented system for physical examination of the locomotor system in occupational medicine (*fokus*^*(C)*^)

**DOI:** 10.1186/1745-6673-2-12

**Published:** 2007-10-29

**Authors:** Michael Spallek, Walter Kuhn, Sieglinde Schwarze, Bernd Hartmann

**Affiliations:** 1 Volkswagen Commercial Vehicles, Health Service, P.O. Box 21 05 80, 30405 Hannover, Germany; 2 lnstitute of Occupational and Social Medicine, Heinrich-Heine-University Duesseldorf, Universitaetsstraße 1, 40225 Duesseldorf, Germany; 3 Bau-Berufsgenossenschaft Hamburg, Holstenwall 8-9, 20355 Hamburg, Germany

## Abstract

Occupational physicians are very often confronted with questions as to the fitness of the postural and locomotor systems, especially the spinal column. Occupational medical assessment and advice can be required by patients with acute symptoms, at routine check-ups, by persons who have problems doing certain jobs, and for expert medical reports as to the fitness of persons with chronic disorders or after operations. Therefore, for occupational medical purposes a physical examination must aim primarily to investigate functions and not structures or radiologic evidence. The physical examination should be structured systematically and according to regions of the body and, together with a specific (pain) anamnesis should provide a basis for the medical assessment.

This paper presents a function-oriented system for physical examination of the locomotor system, named *fokus*^(*C*) ^(**F**unktions**o**rientierte **K**oerperliche **U**ntersuchungs**s**ystematik, also available on DVD). *fokus*^(*C*) ^has been developed with a view to its relevance for occupational medical practice and does not aim primarily to provide a precise diagnosis. Decisive for an occupational medical assessment of disorders of the musculoskeletal system is rather information about functional disorders and any impairment of performance or mobility which they can cause. The division of the physical examination into a rapid **screening **phase and a subsequent more intensive **functional diagnostic **phase has proved its practicability in many years of day-to-day use. Here, in contrast to the very extensive measures recommended for orthopaedic and manual diagnosis, for reasons of efficiency and usability of the system in routine occupational medical examinations the examination is structured according to the findings. So it is reduced to that which is most necessary and feasible.

## 1. Background

Problems with the locomotor system play an important role in the day-to-day work of the occupational physician, in particular with respect to the physical capacity and fitness for work of employees. For the occupational physician, the medical assessment is much more straightforward if the physical examination concentrates systematically on functions rather than on structures and radiologic evidence, and is combined with a well-targeted (pain) anamnesis [1-3]. Many years of practical experience and the limited time allowed for occupational medical examinations speak for a systematic subdivision of the physical examination into a screening phase and, based on the results of this, a subsequent functional diagnostic phase [4]. Depending on the nature of the medical problem or on whether one part of the body is subject to particular strain, these examinations may be carried out as part of a total physical examination or may be restricted to individual regions of the body.

The function-oriented system for physical examination (*fokus*^(*C*)^) makes use of

• a targeted anamnesis (as published in [1]) combined with

• the screening examination

to be sure of recognizing any functional disorders or diseases which are relevant for occupational medicine and, in addition, of the

• more comprehensive functional diagnostic examination to provide a systematic method of searching for functional disorders, deficits or symptoms of disease

(additional files 1, 2, 3).

The results are documented on the basis of the neutral-zero method, comparing both sides of the body and, if necessary, with details of pain reported during the examination. If no abnormalities are detected in anamnesis and screening, there is no compelling reason to carry out further functional diagnostic examinations.

The *fokus*^(*C*) ^system allows the occupational physician to recognize functional, muscular or other deficits of the locomotor system which are relevant at the workplace and also to recognize relevant orthopaedic syndromes as well as the associated differential diagnoses in the symptoms and findings [5-7]. In contrast to the very extensive measures recommended for orthopaedic and manual diagnostics, here for reasons of efficiency and usability of the system in routine occupational medical examinations, the measures are chosen according to the situation and so are reduced to that which is most necessary and feasible.

## 2. Spinal column

### 2.1. Screening of the cervical spine

For the examination of the cervical spine, the patient sits on the examination couch with the back of his (or her) knees at the edge of the couch. During screening the examining physician stands a short distance in front of the patient, during the functional diagnostic examination behind the patient. The screening of the cervical spine includes inspection for postural anomalies, abnormalities, asymmetry, etc. and also active demonstration of cervical mobility by the patient in all directions (additional file 1).

The patient, sitting with the head erect (starting position), is asked to

• turn the head actively as far as possible to both sides (rotation in neutral position); then, again from the starting position, to

• bend the head actively to both sides (lateral flexion); and finally from the starting position

• extension and flexion are tested.

### 2.2. Functional diagnostic examination of the cervical spine

**Figure 1:**
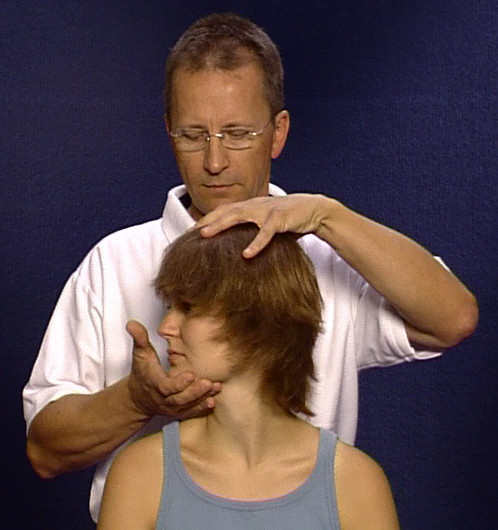
Rotation test for the cervical spine.

If the anamnesis or screening reveal abnormalities, the patient is subjected to a passive, more differentiated testing of the functions of the cervical spine by the examining physician (additional file 1). One hand of the physician fixes the axis of rotation on top of the head, the other hand pulls the chin in the direction of movement (figure 1). First the amount of possible rotation from neutral position is checked, for both sides, until the functional or pain-induced end of the movement. The test for rotation in neutral position is then followed by further rotation tests, always comparing the two sides,

• in anteflexion (tests mainly mobility of the skull joints) and

• in retroflexion (tests mobility of the middle and lower cervical spine).

These mobility tests should always be ended with a slight springing back movement so that an impression of the mobility at the end of the range is obtained: a hard end of the movement suggests that it is limited by bone, a soft end suggests that the movement is limited by soft tissue (e.g. shortened muscle, etc.).

• the test for lateral bending involves pressure of the examiner's hand on the side of the head in the direction of movement with the other hand positioned on the trapezius muscle on the other side to register evasive movements.

• The maximum extension and flexion of the cervical spine is recorded in terms of the chin-jugulum distance in cm.

• This is followed by percussion tests and tests for tenderness to pressure over the spinous processes of the cervical spine down to the cervicothoracic boundary, then on both sides of the skull cap along the nuchal line, on the upper medial angle of the scapula (origin of the musculus levator scapulae) and on the lateral upper margin of the scapula for the m. trapezius.

The tests for compression and traction of the neck are used to distinguish cervical spine disorders originating in the intervertebral discs or facets from mainly muscular problems [8]. For both of these functional tests, the examiner stands behind the patient at the edge of the couch and holds the upper part of the patient's body against his own body to give the patient a secure feeling and also to be able to register any evasive movements in the dorsal direction.

**Figure 2:**
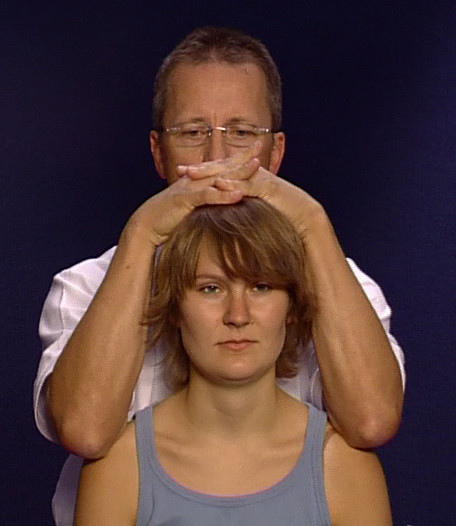
Cervical compression test.

• The neck compression test takes place with the hands of the examiner on top of the patient's head cautiously exerting increasing caudal pressure; the examiner's elbows should remain in contact with the patient's shoulders (Figure 2). Sudden jerks should be avoided. In this test the foramina intervertebralia are narrowed and the facets of the cervical vertebrae moved towards each other. In this way, radicular symptoms can be provoked in persons with intervertebral disc problems. From their position at the end of the neck compression test, the hands of the examiner move to the temporal region with the thumbs under the skullcap on the mastoid process.

• Symmetrical vertical cranial traction of the neck follows. This relieves the pressure on the intervertebral joints and stretches passively the generally shortened muscles of the cervical spine.

In addition the biceps, triceps and brachioradialis reflexes should be tested and sensitivity should be checked in the cervical spine dermatomes. If there are indications of a possible vascular problem or of a thoracic outlet syndrome, an Adson's test may be considered [8].

Differential diagnostic information yielded by this examination includes evidence of a cervicobrachial syndrome or of pseudoradicular symptoms and of a cervicocephalic syndrome or, by exclusion diagnosis, of a cervical syndrome (table 1).

**Table 1:** Differential diagnosis of cervical spine disorders

Cervical syndrome
Cervicocephalic syndrome
Cervicobrachial/radicular syndrome
disorders affecting spinal segment C5
disorders affecting spinal segment C6
disorders affecting spinal segment C7
disorders affecting spinal segment C8
disorders affecting spinal segment Th1

### 2.3. Screening of the thoracic and lumbar spine and the lumbar-pelvic-iliac region

Screening of the thoracic and lumbar spine and the lumbar-pelvic-iliac region is carried out with the patient standing, as it is the first part of the functional diagnosis. For the second part of the functional diagnosis, the patient lies down.

For the screening, the examining physician sits behind the patient who is standing undressed. This is an optimal position for inspection of the waist indentations, the trochanters and malleoli, the trapezium contours and scapula position and most especially for inspection of the pelvis because the eyes of the sitting examiner are at about the height of the patient's iliac crest. In addition, this position of the examiner makes it possible for him to check and perhaps correct the way the mobility tests are carried out.

The screening begins with (additional file 1)

• the test of flexion, determined as finger-floor distance (cm). This gives a first impression of the overall mobility of the thoracic and lumbar spine and the hips. In the subsequent

• test for lateral bending, first continuous and discontinuous bending of the thoracic and lumbar spine are compared and then the distance of the fingertips to the lateral side of the knees registered for both sides. With

• the sideways rotation test, preferably with the hands clasped behind the head and perhaps with fixation of the pelvis by the examiner, discrete scoliosis becomes more evident. This is followed by percussion and tenderness tests for the spine, the iliolumbar ligament and the sacroiliac joint.

The tests for standing on the heels and toes and for walking on the heels (L4/L5) and toes (S1) can provide evidence of radicular disorders.

**Figure 3:**
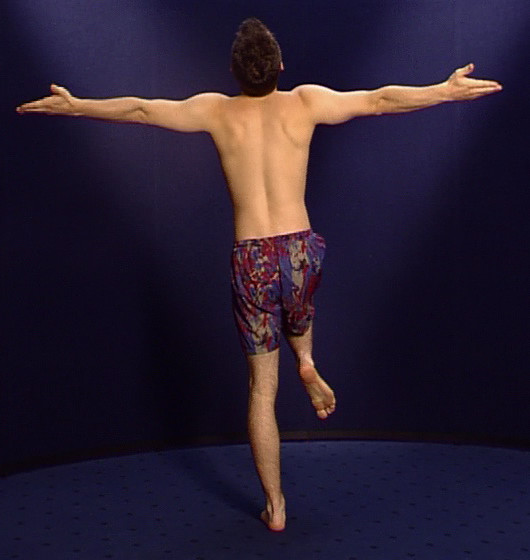
Balancing on one leg.

This is followed by balancing on one leg, preferably on the so-called standing leg, with arms stretched out, palms of the hands upwards, head up to the ceiling and eyes closed (Figure 3). A period balancing on one leg of <6 seconds (after at least 3 tries) can be evidence of expected back pain and an indicator of coordination disorders which often precede clinical symptoms [9,10].

The last test, squatting with the heels on the floor and standing up from the squat, yields not only evidence of shortened muscles, e.g. the triceps muscle of the calf, but also information about the harmony of the whole series of motions [3,11].

### 2.4. Functional diagnostic examination of the thoracic and lumbar spine and the lumbar-pelvic-iliac region

If the anamnesis is indicative of problems or the results of the screening are abnormal, a functional diagnostic examination should follow (additional file 1). In the first part the examiner is behind the standing patient. The flexion test of the spine is repeated and followed by an extension test.

**Figure 4:**
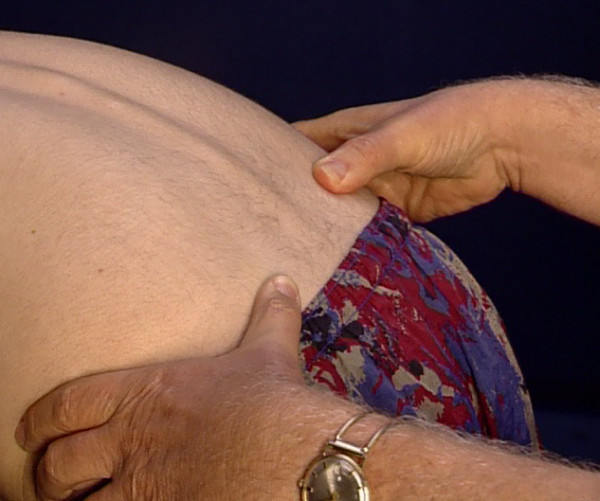
Testing for cranial movement of the spinae iliacae posteriores superiores.

In the flexion test, the examiner places his thumbs on the spinae iliacae posteriores superiores, one on each side, and monitors especially cranial movement of these spinae with different degrees of flexion of the patient [12] (Figure 4). This can yield evidence of disorders or blockages in the sacroiliac joint. In addition, the mobility test of Ott for the thoracic spine and the technique of Schober for mobility of the lumbar spine should be carried out in anteflexion and retroflexion.

The second part of the functional diagnostic examination should be carried out if possible on a couch which can be approached by the examiner from all sides. The patient is supine. This part of the functional diagnostic examination begins with

**Figure 5:**
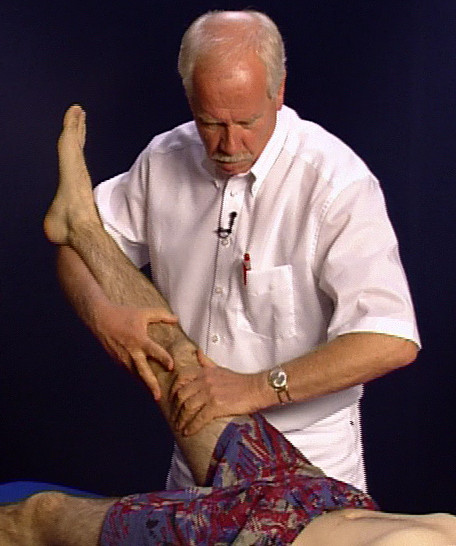
The Lasègue test.

• the isometric testing of resisted movement of the long extensor muscle of the great toe on both sides, a muscle supplied from segment L5.

• The isometric eversion test of the foot tests the muscles of the peroneus group which are supplied from segment S1.

• Mainly the anterior tibial muscle is tested by the inversion test as evidence of disorders in Segment L4.

• Absence or marked weakening of the Achilles tendon reflex suggests a disorder in Segment S1.

• The same symptoms for the patellar tendon reflex are evidence of a disorder in Segment L4.

• The Lasègue sign yields evidence of several different potential diagnoses if the test is carried out as shown in Figure 5 with passive flexion of the supported leg: intradiscal displacement, disc prolapse, Coxarthrosis, shortening of the ischiocrural musculature, symptoms of meningeal irritation, blockages in the sacroiliac joint, psychogenic overlay [13].

• In addition, the Bragard test for nerve root irritation is carried out.

From this position of examiner and patient the flexion test of the hip joint can readily be carried out. The test for hip joint extension can be omitted because it would require turning the patient into the prone position and because it contributes little towards the recognition of the capsular pattern of the hip joint [14]. The subsequent test of medial rotation is the most important test for disorders of the hip joint. Abnormalities of lateral rotation are more indicative of periarticular problems. Both tests are finished at the end of the range of movement with a slight, gentle springing back movement.

The subsequent hyperabduction test of Patrick (sign of four) [15] yields

• information about the ipsilateral hip joint and sacroiliac joint if the opposite side of the pelvis is held still and

• information about the facet joints of the spine if no pressure is put on the pelvis [12,16].

These tests too are finished with a slight springing back movement. Finally an adduction test of the hip joint is carried out as additional information about the sacroiliac joint and sensitivity tests on the various dermatomes for additional differentiation between radicular and pseudoradicular symptoms.

Confirmatory tests complete and extend the functional diagnostic examination:

• In the supine long sitting test the patient is asked to sit up from a lying position and to indicate where pain from the back radiates into the leg. The results of the long sitting test should, for example, correspond with those from the flexion test.

• In the reclination test the patient sits with the backs of his knees against the end of the couch. With one hand the examiner holds one of the patient's thighs proximal to the patella and with the other hand brings the lower leg on the same side into a stretched position. In this test the two sides must be compared. A typical evasive movement, for example, kyphosis of the thoracic spine when the lower leg is stretched, can be confirmation of a form of sciatic pain or radicular disorder and so should correspond with the results of the Lasègue test [4,15].

The differential diagnostic evidence which may be obtained from the examination of the spine is summarized in table 2.

**Table 2:** Differential diagnosis of lumbar spine disorders

Lumbar pain
Lumbago
Sciatica
Radicular syndrome
disorders affecting spinal segment L4
disorders affecting spinal segment L5
disorders affecting spinal segment S1
Sacroiliac joint symptoms
Coxarthrosis/coxalgia

## 3. Shoulder-arm region

### 3.1. Screening of the shoulder-arm region

The examination of the shoulder-arm region begins with inspection (additional file 2).

• In the functional test for the degree of abduction and external rotation of the shoulder and the flexion of the elbow joint the patient places his hands, one at a time, at the back of his neck with the thumb pointing downwards. The caudally abducted thumbs make it easy to recognize lateral differences.

• The functional test for adduction and internal rotation of the shoulder joint and flexion in the elbow involves asking the patient to place his hands behind his back (in the position to tie an apron), one at a time, with the thumb pointing upwards.

In these two tests, differences of as much as 2 cm between the two sides can be considered normal.

**Figure 6:**
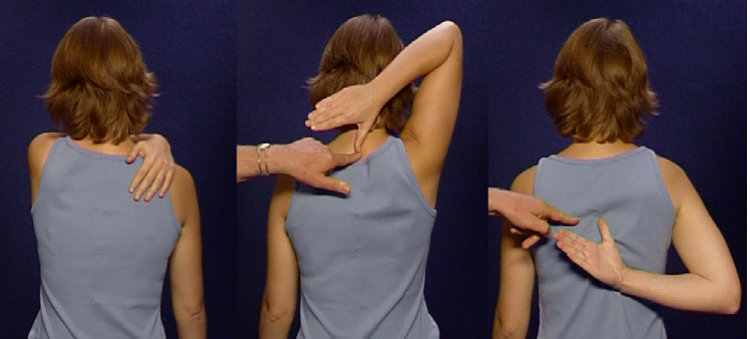
Screening shoulder and arm function: patient's hand at the back of her neck, behind her back and on her opposite shoulder.

Asking the patient to place his hand on the opposite shoulder tests the adduction in the shoulder joint and the mobility of the acromioclavicular and sternoclavicular joints. This simple and rapid shoulder screening covers all the functions of the shoulder-arm region which are relevant for occupational medicine (Figure 6). If the anamnesis, inspection and screening in this form have revealed no abnormalities, no functional disorders of relevance for occupational medicine are present

### 3.2. Functional diagnostic examination of the shoulder-arm region

The functional diagnostic examination of the sitting patient begins with the active abduction and elevation of the arm, if possible to 180° (additional file 2). This can reveal a "painful arc" (typically between 70° and 120°) which is indicative of a disorder in the region of the bursa subacromialis and the neighbouring area. This is followed by a passive elevation test in which the examining physician raises the gently extended arm from the shoulder joint. In this way the mobility test in abduction and elevation is carried out without strain on the bursa subacromialis and so in a typical case of "painful arc" the movement would cause less pain.

The most reliable test of internal and external rotation is carried out with the arms horizontal (90° abduction of the shoulder and 90° flexion of the elbow joint) by rotating the forearm upwards and downwards.

In the test of the acromioclavicular joint [17] the arm of the patient is pulled towards the opposite shoulder to bring the joint under compression on the arm side.

**Figure 7:**
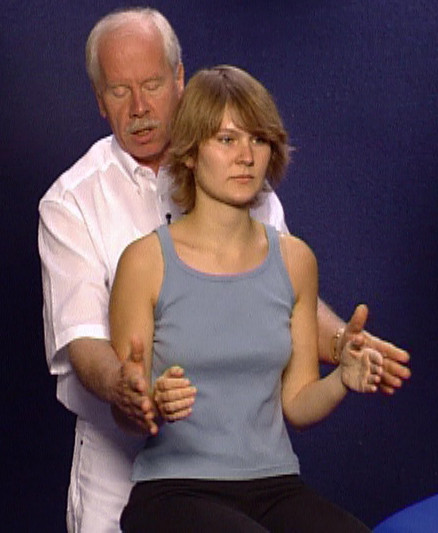
Position of the examiner for isometric testing of m. infraspinatus

**Figure 8:**
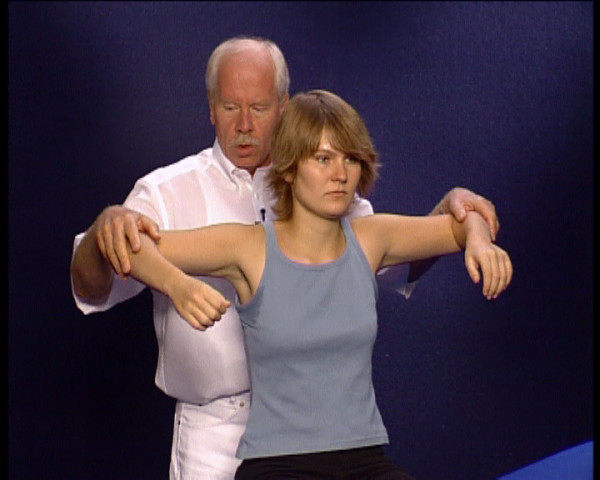
Position of the examiner for isometric testing of m. deltoideus.

This is followed by isometric functional tests of the shoulder girdle and muscles of the upper arm. The examiner stands behind the patient and begins by fixing the patient's upper arms against the patient's body in order to prevent evasive movements during the rotation tests (Figure 7). In sequence the following isometric functions are tested:

• the external and internal rotation of the shoulder with the upper arm against the body (musculus infraspinatus and m. subscapularis)

• forearm flexion from the elbow in supination, semipronation and pronation (m. biceps, m. brachioradialis, m. brachialis)

• abduction of the upper arm to the 30° position (m. supraspinatus) and to over 70° (m. deltoideus) (Figure 8), and

• adduction of the upper arm (m. pectoralis major, m. latissimus dorsi).

This extensive isometric functional diagnostic examination, together with the anamnesis and with reference to the typical capsular patterns of shoulder disorders [14], makes relatively simple the diagnosis, on the one hand, of freezing arthritis, for which the symptoms were described by Wagenhaeuser and Mumenthaler as long as 30 years ago [18,19], and, on the other hand, of the various more recently described impingement syndromes (Reichelt in [6]) and the differential diagnosis when rotator cuff rupture is suspected [6,20].

## 4. Arm-hand region

### 4.1. Screening of the arm-hand region

For the screening of the arm-hand region for occupational medical purposes it is sufficient, after anamnesis and inspection, to carry out a test in which the patient actively grips and lifts a chair by the back with the hands in pronation and supination position ("Chair-Test" [12], (additional file 2). Especially in persons with epicondylar pain or problems with the wrist or tendon sheaths abnormal reactions are to be expected in this test.

For persons whose work involves hand-arm vibration exposure the screening can be extended by asking the patient to support himself on his wrists during maximum extension and flexion.

### 4.2. Functional diagnostic examination of the arm-hand region

• If a functional diagnostic examination is necessary, a series of movements which are relevant to the person's work are carried out actively (that is, the patient carries out the movements) and also isometrically against resistance provided by the examining physician (additional file 2). Tested should be the ranges of movement of

• the forearm in pronation and supination,

• dorsal extension (mediocarpal joint) and palmar flexion (radiocarpal joint), and

• ulnar and radial duction in the region of the wrist. Then follows

• the test for mobility and strength during finger abduction and adduction by spreading out and closing together of the fingers and

• during opposition of the thumb to each of the fingers (to form a ring). In addition,

• the extension of the thumb against resistance is tested.

During the opposition test the examiner should try to break open with his fingers each ring formed with the patient's thumb and finger. Especially during opposition of the thumb and the ring finger, this test can be helpful in determining the relevance of tennis elbow symptoms.

For persons exposed to hand-arm vibration, it is recommended that particular attention be given to the maximum range of active and passive movement and to pain provocation in the wrist.

## 5. Knee-ankle region

### 5.1. Screening of the knee-ankle region

The screening of the functional capacity of the knee and ankle joints takes place for both regions at once with the patient standing. It includes examination modules which are also used for screening of the spine (additional file 3).

• After inspection of the area, screening tests of mobility, strength and coordination of the lower extremities are carried out. They begin with the patient squatting as low as possible with his heels on the floor and subsequently standing up slowly.

• The next test, balancing on one leg – right and left – is followed by asking the patient to hop briefly on one leg – right and left – and also to

• make a few steps walking on his toes and then on his heels (cf. Section 2.3) With the request that the patient stand on the outer edges of his feet, the screening is complete.

If the anamnesis, inspection and screening in this form have revealed no abnormalities, no functional disorders of relevance for occupational medicine are present.

### 5.2. Functional diagnostic examination of the knee-ankle region

The functional diagnostic examination of the lower extremities is carried out separately for the knee and ankle regions (additional file 3). also because occupational medical questions as to the fitness of this region involve the knee joint much more frequently than the ankle [1].

#### 5.2.1. Knee

The examination of knee joint function takes place passively for the supine patient and begins with

• palpation of the kneecap (especially tenderness to pressure, whether it can be displaced, perhaps effusion, etc.) and

• the inner and outer ligaments of both knees.

• The functional test of extension and flexion of the knee joints is followed by a

• test of stability of the collateral ligaments by checking the range of movement of the lower leg relative to the upper leg with the knee bent by 20° (valgus and varus stress).

• Twisting the lower leg with the knee bent by 90° tests not only the range of internal and external rotation but also the intactness of the medial or lateral meniscus (Steinmann's sign [12,21].

• The integrity of the anterior cruciate ligament is tested by means of the so-called Lachmann test. The knee joint is bent to an angle of about 15° to 30° and the patient relaxes his thigh muscles; the examiner fixes the femur with one hand and pulls the tibia forward with the other. A soft end point or lack of a stop at the end of the range of movement ("drawer") is evidence of a lesion of the cruciate ligament [22].

For reasons of practicability, occupational medicine dispenses with the large number of similar functional tests (e.g. the stable Lachmann test, the no-touch Lachmann test, etc.) because abnormal results in the functional diagnostic examination should be followed by an orthopaedic or traumatological examination.

A supplementary part of the meniscus examination (see Steinmann's sign I) is not carried out until after the Lachmann test; it involves

• Apley's distraction and compression tests [12] and is postponed until now because the patient must turn over into the prone position. With the patient's knee bent (90°) the examiner fixes the thigh of the same leg on the couch with his own knee and subjects the patient's lower leg to internal and external rotation while distraction and grinding. Pain under compression is also indicative of meniscus damage; it should become less under traction.

• The final test of the musculus quadriceps takes place with the patient sitting on the edge of the couch. The patient stretches his leg against the resistance from the examiner's hand on the lower leg and tries actively to hold the stretched end position against the pressure.

#### 5.2.2. Ankle joint

• In addition to the screening tests, the so-called anterior talus test is carried out to check the anterior talofibular ligament. The heel of the ankle being examined rests on the fist of the examiner who tries, with his other hand, to move the distal lower leg towards the couch.

• This is followed by tests of adduction, inversion and supination for each ankle. By pressing together the inner and outer ankle bones (squeeze test) evidence of the stability of the syndesmosis may be obtained.

• The final test for stability of the calcaneofibular ligament is carried out by percussion of the examiner's fist against the heel of the patient, who is preferably sitting with the lower leg hanging and relaxed (so-called "click test").

## 6. Supplementary notes

The functional diagnostic examinations were put together with regard to an existing functional anamnesis incl. pain and occupational tasks and the following premises :

Optimized organization in 2 steps : screening and function

Module systematic for the body regions : spine, shoulder, arm-hand, knee-ankle

Ergonomic requirements for the patient and the physician

Aims at the most relevant symptoms in occupational medicine

High reliability of the function tests

No X-ray

Simple documentation

The function-oriented system takes into account the fact that occupational medical prophylaxis does not aim primarily at producing a therapeutic indication but at prevention. From the large number of available validated tests for function of the locomotor system a selection was made intentionally and on the basis that the tests are readily learned and used, and that they have proved useful in occupational medical practice. The selected tests had to be sufficiently sensitive in the detection of functional disorders or symptoms which are relevant in occupational medicine. Systematic examination of persons with symptoms affecting the locomotor system is the most important basis for making an objective assessment of functional disorders and limitations and is decisive for a proper medical opinion as to the reasonableness of certain working conditions for an individual employee. Therefore it is an important component of the advice given to employees and employers and plays an important role in the prevention of work-related or work-induced disorders of the locomotor system [4,23].

## Competing interests

The author(s) declare that they have no competing interests.

## Authors' contributions

WK has made substantial contributions to the examination method. All authors have been involved in revising the manuscript critically. All authors have read and approved the final manuscript. An English version of the anamnesis is not yet available at present but is being prepared for submittal to this journal.

## Supplementary Material

Additional file 1 Examination Schedule 1: *fokus*^*(C) *^examination of the spinal column. The table shows the different stages of examination of the spinal column (screening – functional examination) following the fokus^(C) ^schedule. Click here for file

Additional file 2 Examination Schedule 2: *fokus*^(*C*) ^examination of the shoulder-arm region. The table shows the different stages of examination of the shoulder-arm region (screening – functional examination) following the fokus^(C) ^schedule. Click here for file

Additional file 3 Examination Schedule 3: *fokus*^(*C*) ^examination of the knee and ankle. The table shows the different stages of examination of the knee and ankle region (screening – functional examination) following the fokus^(C) ^schedule. Click here for file
