# Supplementary figures and images for: Occupational medical prophylaxis for the musculoskeletal system: A function-oriented system for physical examination of the locomotor system in occupational medicine (fokus(C))
Source: J Occup Med Toxicol. 2007 Oct 29;2:12. doi: 10.1186/1745-6673-2-12 (PMC2174926; doi:10.1186/1745-6673-2-12)

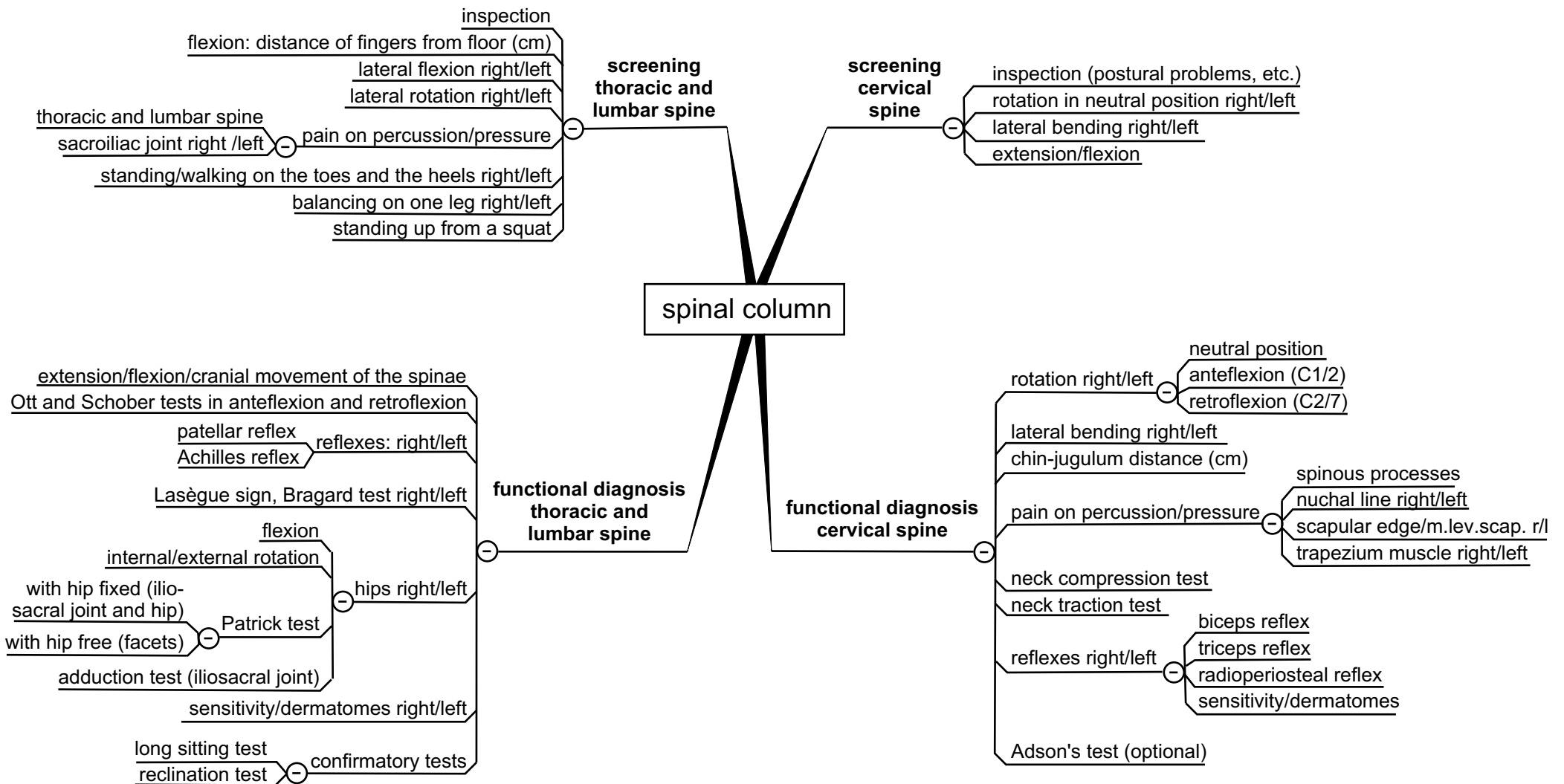

Supplement: Additional file 1 — Examination Schedule 1: fokus(C) examination of the spinal column. The table shows the different stages of examination of the spinal column (screening – functional examination) following the fokus(C) schedule. [file 1745-6673-2-12-S1.pdf]

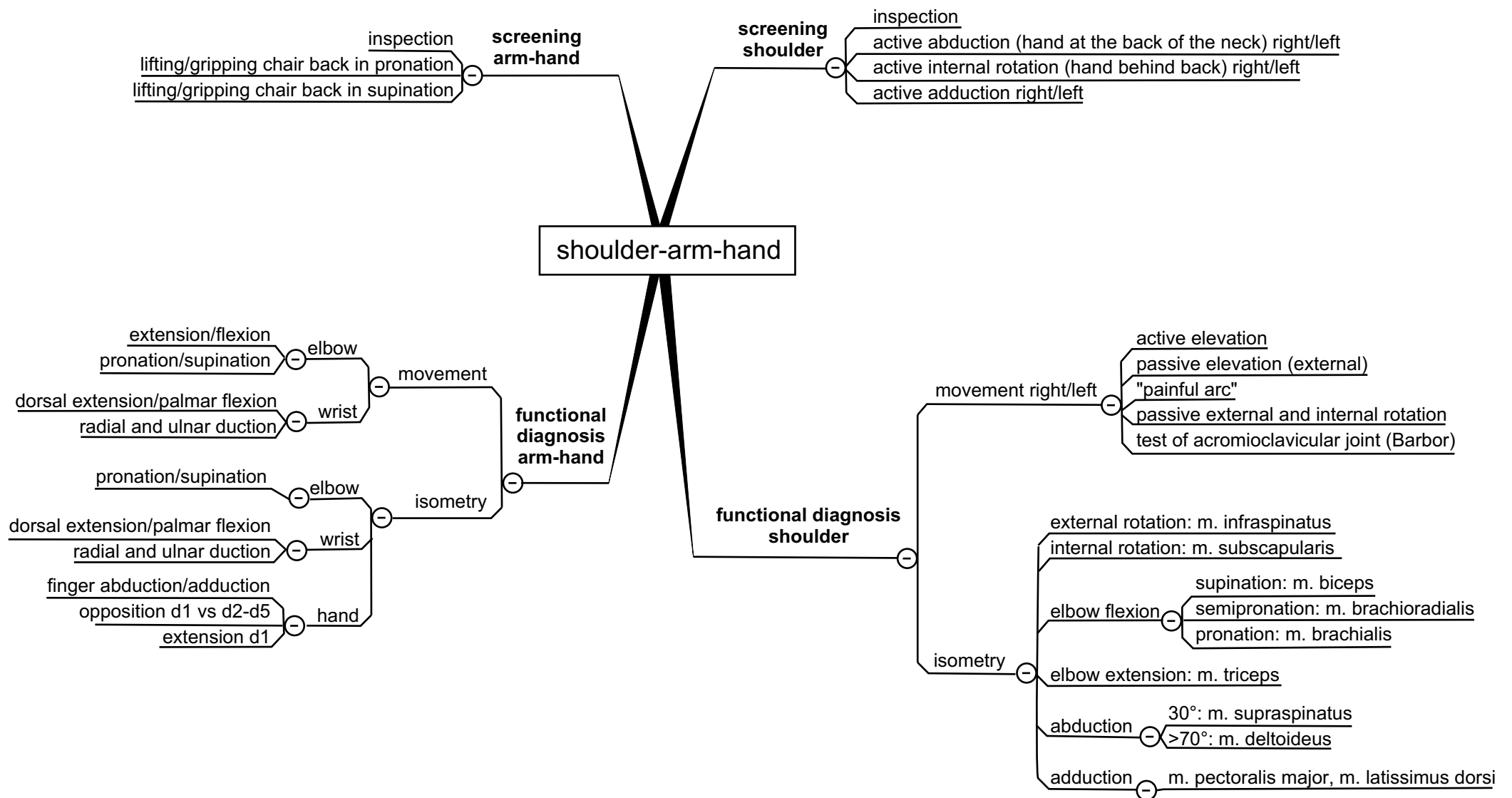

Supplement: Additional file 2 — Examination Schedule 2: fokus(C) examination of the shoulder-arm region. The table shows the different stages of examination of the shoulder-arm region (screening – functional examination) following the fokus(C) schedule. [file 1745-6673-2-12-S2.pdf]

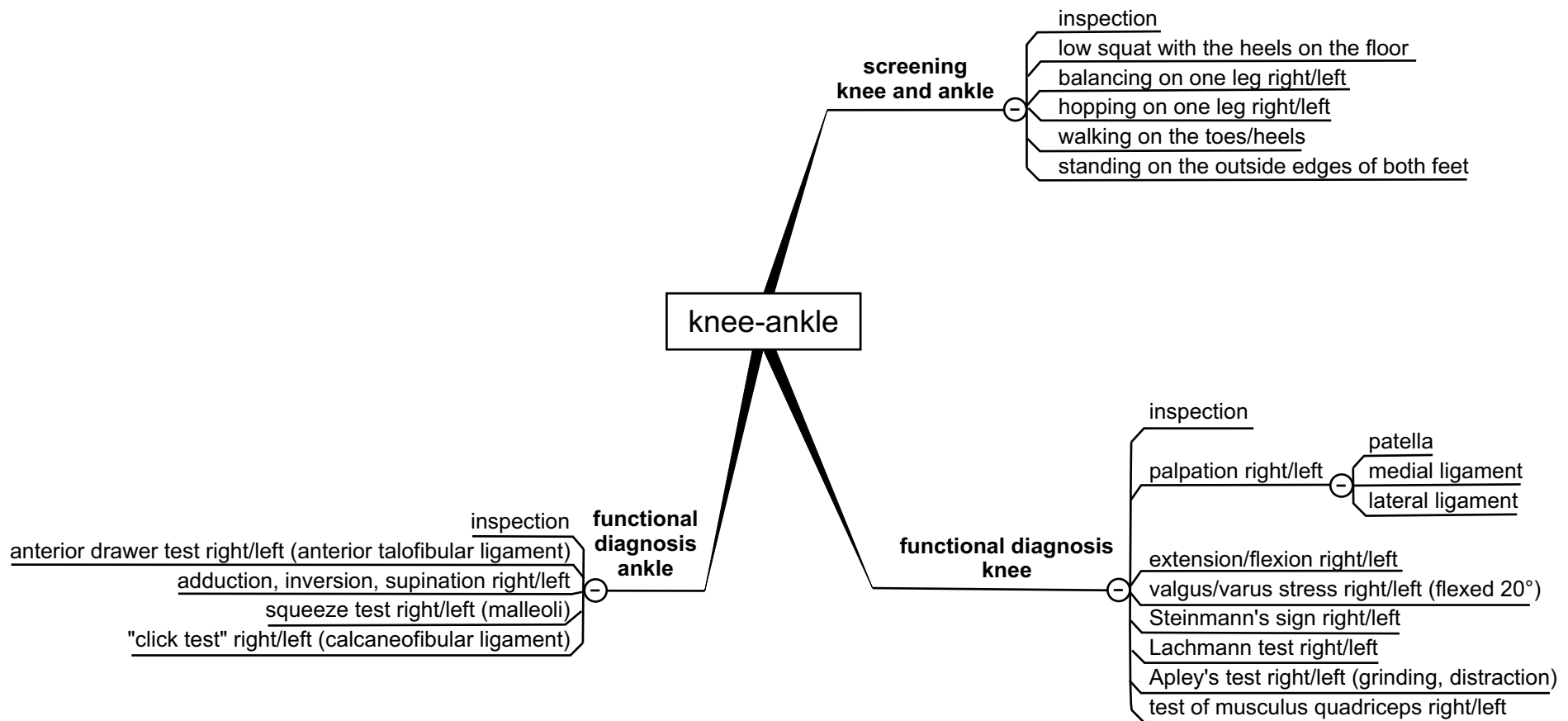

Examination schedule 3: *fokus*© examination of the knee and ankle

Supplement: Additional file 3 — Examination Schedule 3: fokus(C) examination of the knee and ankle. The table shows the different stages of examination of the knee and ankle region (screening – functional examination) following the fokus(C) schedule. [file 1745-6673-2-12-S3.pdf]
